# The TriArtic procedure: TRIcuspid annular reduction by TEER in the commissure—a transcatheter annuloplasty enabling EVOQUE tricuspid valve replacement: a case report

**DOI:** 10.1093/ehjcr/ytag729

**Published:** 2026-10-06

**Authors:** Raphael Phinicarides, Fabian Voß, Tobias Zeus, Amin Polzin

**Affiliations:** Department of Cardiology, Pulmonology, and Vascular Medicine, University Hospital Düsseldorf, Medical Faculty of the Heinrich Heine University Düsseldorf, 40225 Düsseldorf, Germany; Department of Cardiology, Pulmonology, and Vascular Medicine, University Hospital Düsseldorf, Medical Faculty of the Heinrich Heine University Düsseldorf, 40225 Düsseldorf, Germany; Department of Cardiology, Pulmonology, and Vascular Medicine, University Hospital Düsseldorf, Medical Faculty of the Heinrich Heine University Düsseldorf, 40225 Düsseldorf, Germany; Department of Cardiology, Pulmonology, and Vascular Medicine, University Hospital Düsseldorf, Medical Faculty of the Heinrich Heine University Düsseldorf, 40225 Düsseldorf, Germany; Cardiovascular Research Institute Düsseldorf (CARID), Medical Faculty, Heinrich-Heine University, 40225 Düsseldorf, Germany

**Keywords:** Atrial functional tricuspid regurgitation, Case report, EVOQUE, Sequential transcatheter therapy, Transcatheter annuloplasty, Transcatheter tricuspid valve replacement, TriArtic procedure, TriClip

## Abstract

**Background:**

Transcatheter tricuspid valve replacement (TTVR) with the EVOQUE system has expanded therapeutic options for severe tricuspid regurgitation (TR), but a prohibitively dilated tricuspid annulus may exceed the device-sizing matrix and preclude implantation. No transcatheter strategy has been described to mechanically pre-condition such an annulus.

**Case summary:**

A 71-year-old woman with heart failure with reduced ejection fraction (left ventricular ejection fraction 38%, suspected premature-ventricular-complex–induced cardiomyopathy), permanent atrial fibrillation, and class III obesity presented with torrential atrial functional TR refractory to fully optimized guideline-directed medical therapy. Screening cardiac computed tomography (CT) classified the native annulus as ‘outside parameters’ for the 56 mm EVOQUE (Edwards Lifesciences); the Heart Team also deemed standalone tricuspid edge-to-edge repair (T-TEER) inadequate owing to the massively dilated annulus. We performed the TriArtic procedure—a single TriClip XTW (Abbott) deployed in the septo-anterior position as a transcatheter annuloplasty step. Repeat CT after 27 days demonstrated an ∼11% reduction in tricuspid annular area and conversion of projected-derived-diameter oversizing from −2.9% to +3.3% (within parameters). Transfemoral EVOQUE 56 mm was implanted 48 days after the TriClip; postprocedural imaging confirmed no residual regurgitation and a mean transtricuspid gradient of 1 mm Hg, with rapid recovery of cardiorenal function.

**Discussion:**

Transcatheter tricuspid annuloplasty by a strategically placed commissural TEER device may convert a prohibitively dilated annulus into one within EVOQUE-implantable parameters. In carefully selected patients ineligible for either standalone strategy, sequential transcatheter annuloplasty followed by valve replacement—the TriArtic procedure—may offer a feasible pathway, conceptually analogous to the mitral ARCTIC technique. This single case demonstrates feasibility only and does not establish general applicability.

Learning Points

- A tricuspid annulus too large for EVOQUE yet too dilated for durable standalone TriClip repair has no standalone transcatheter option.
- A single commissural TriClip can downsize the annulus, converting an ‘outside-parameters’ CT screen into EVOQUE-implantable anatomy.
- This off-label staged strategy rests on a small, volume-dependent oversizing margin—requiring euvolaemia, Heart Team review, consent, and close imaging follow-up.

## Introduction

Transcatheter tricuspid valve replacement (TTVR) with the EVOQUE system (Edwards Lifesciences) has recently entered routine clinical care for selected patients with severe symptomatic tricuspid regurgitation (TR), with the TRISCEND II trial demonstrating superior reduction in TR severity and quality-of-life benefit compared with medical therapy alone.^1^ Tricuspid edge-to-edge repair (T-TEER) with the TriClip system (Abbott) remains the most widely used transcatheter approach for severe atrial functional TR, supported by the TRILUMINATE Pivotal 1- and 2-year outcomes.^2,3^ However, a subset of patients displays a tricuspid annulus that is at once too large for current TTVR sizing matrices and too dilated for durable clip-based coaptation, leaving no standalone procedural option. In this case report, we describe a staged strategy—which we provisionally term the TriArtic procedure (TRIcuspid Annular Reduction by TEER In the Commissure)—in which a single TriClip is deployed primarily as a transcatheter annuloplasty step, converting a screen-failed annulus into one that meets EVOQUE oversizing thresholds. The concept is a tricuspid analogue of the ARCTIC procedure described by Condos *et al*. for valve-in-mitral-annular-calcification transcatheter mitral valve replacement.^4^

## Summary figure

**Figure:**
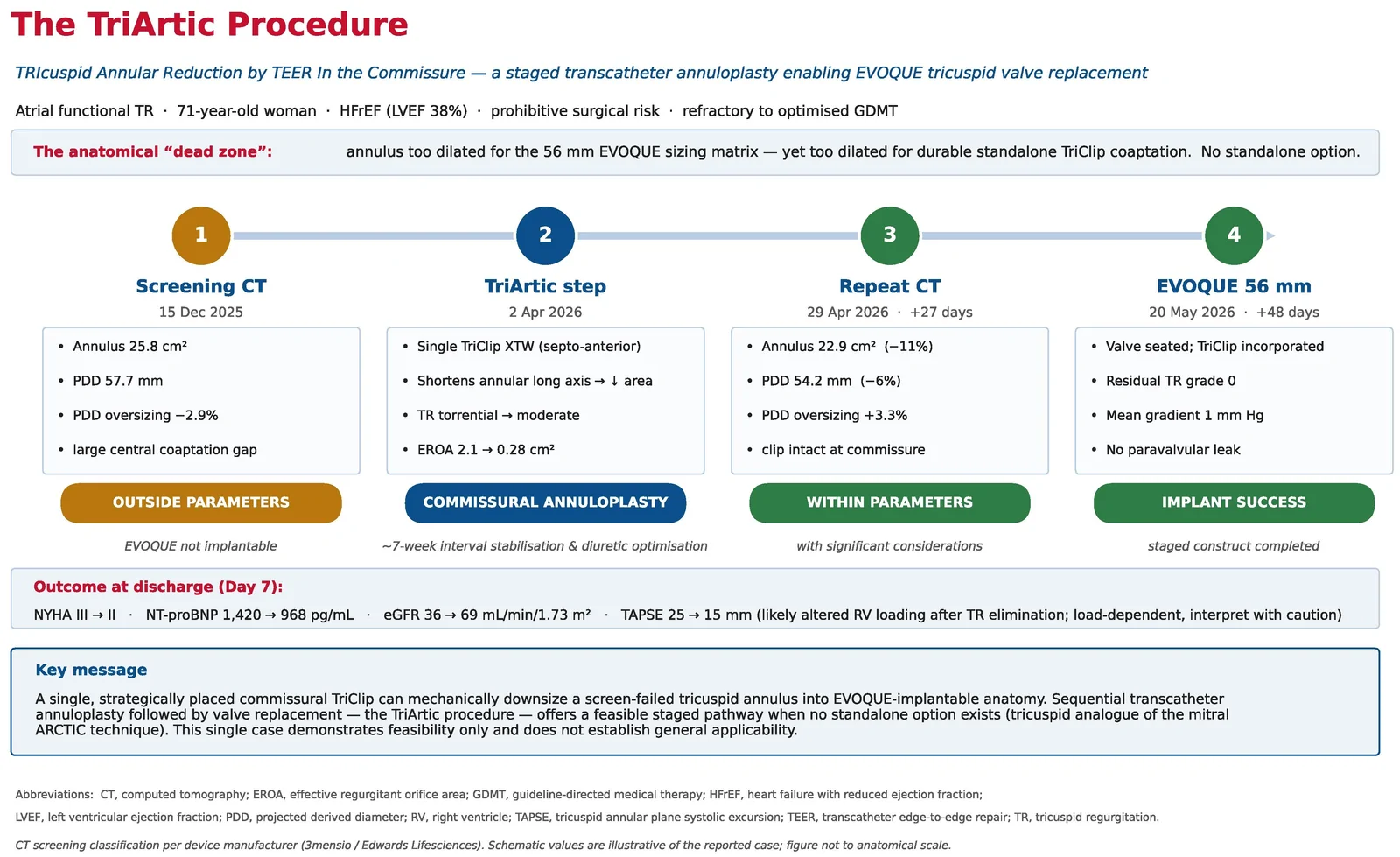


## Case presentation

A 71-year-old woman (height 175 cm, weight 117 kg, body-surface area 2.3 m^2^; class III obesity) presented with progressive exertional dyspnoea, peripheral oedema, and recurrent congestive decompensations despite optimized guideline-directed heart-failure therapy comprising sacubitril/valsartan, bisoprolol, spironolactone, and empagliflozin, together with an oral loop diuretic (furosemide 40 mg once daily, torasemide-equivalent). Further loop-diuretic escalation was precluded by symptomatic hypotension and a reproducible rise in serum creatinine on up-titration; the dose therefore represented the maximally tolerated rather than a ceiling diuretic dose in this forward-failure/cardiorenal physiology. Her clinical status was New York Heart Association (NYHA) class III. Comorbidities included permanent atrial fibrillation on apixaban, heart failure with reduced ejection fraction (HFrEF) with a left ventricular ejection fraction (LVEF) of 38% with an asynchronous contraction pattern and suspected premature-ventricular-complex–induced cardiomyopathy, COPD GOLD II, lipoedema, and substituted hypothyroidism. Surgical risk was elevated (EuroSCORE II 6.06%, ASA class III).

Baseline transthoracic and transoesophageal echocardiography (20 February 2026) confirmed torrential atrial functional TR (effective regurgitant orifice area [EROA] 2.1 cm^2^, regurgitant volume 160 mL, vena contracta 19 mm), severe biatrial dilatation, a dilated right ventricle (RV) with preserved systolic function (Tricuspid Annular Plane Systolic Excursion - TAPSE 25 mm), and a dilated inferior vena cava with absent respiratory variation (respiratory-fixed; <50% inspiratory collapse), consistent with elevated right atrial pressure. Concomitant moderate mitral regurgitation (EROA 0.16 cm^2^, vena contracta 4 mm), mild aortic regurgitation, and mild pulmonary regurgitation were noted.

Right-heart catheterization (2 April 2026, via right radial and right femoral access) demonstrated a mean right atrial pressure of 6 mm Hg, pulmonary artery pressure of 37/8 mm Hg (mean 19 mm Hg), a directly measured left ventricular end-diastolic pressure of 11 mm Hg (used as the left-heart filling-pressure surrogate, as pulmonary capillary wedge pressure was not separately transduced), pulmonary vascular resistance of 292 dyn·s·cm^−5^ (3.65 Wood units), and cardiac index of 2.25 L·min^−1^·m^−2^. Taken together, these values indicate the absence of significant pulmonary hypertension and no relevant post-capillary (pulmonary venous) component—the normal left-sided filling pressure excludes isolated or combined post-capillary physiology—so that the haemodynamic burden was attributable predominantly to the torrential TR itself. Coronary angiography excluded relevant epicardial disease. Baseline renal function was preserved to mildly impaired (serum creatinine 0.78–0.96 mg/dL, estimated glomerular filtration rate - eGFR 59–77 mL·min^−1^·1.73 m^−2^), without overt cardiorenal syndrome at baseline. Baseline N-terminal pro–B-type natriuretic peptide (NT-proBNP) was 1256 pg/mL and fell to 996 pg/mL after outpatient diuretic optimization, indicating that the patient was assessed in a relatively compensated, near-euvolaemic state rather than during acute volume overload; the recurrent decompensations were interspersed with compensated intervals, and this moderate absolute NT-proBNP is consistent with chronic compensated right-sided congestion in the setting of permanent atrial fibrillation and marked atrial dilatation. Chronic γ-glutamyltransferase elevation (164 U/L) indicated congestive hepatopathy; bilirubin was normal (*Figure 1*).

**Figure 1:**
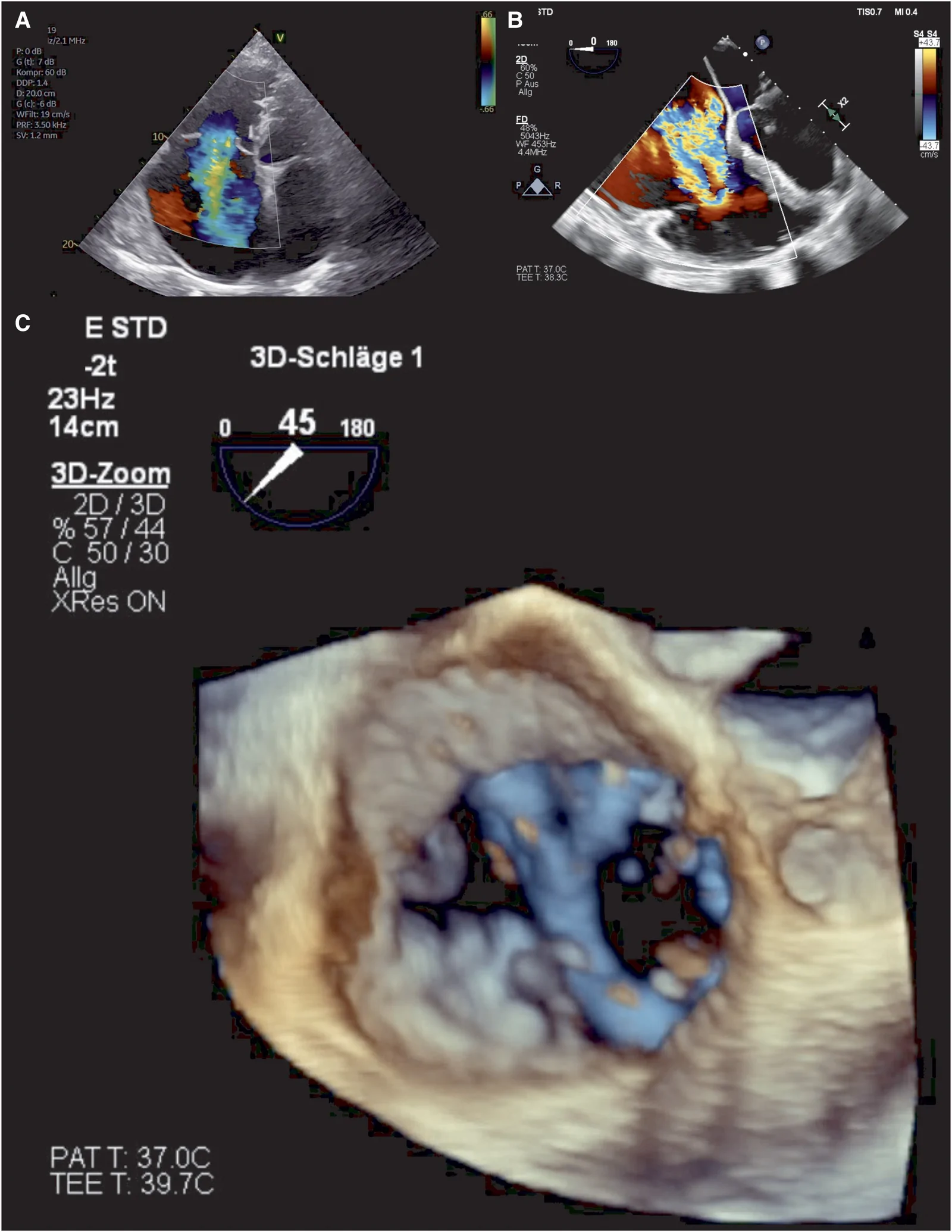
Baseline echocardiography. Baseline imaging (20 February 2026). (*A*) Transthoracic colour-Doppler echocardiography showing a torrential tricuspid regurgitant jet with a broad vena contracta filling the right atrium. (*B*) Transoesophageal colour-Doppler view confirming the wide regurgitant jet. (*C*) 3D transoesophageal en face view of the tricuspid valve demonstrating marked annular dilatation with a large central coaptation gap. Effective regurgitant orifice area 2.1 cm^2^, regurgitant volume 160 mL, vena contracta 19 mm. RA, right atrium; TR, tricuspid regurgitation.

Dedicated EVOQUE screening computed tomography (CT) (15 December 2025) demonstrated a tricuspid annulus that exceeded the sizing window of the largest available (56 mm) EVOQUE valve (annulus area 25.8 cm^2^; projected derived diameter [PDD] 57.7 mm in diastole/58.1 mm in systole; basal-RV short-axis diameter 71.7/62.3 mm). The EVOQUE system is supplied in 44-, 48-, 52-, and 56-mm sizes; secure radial anchoring requires the selected valve to be larger than the native annulus—that is, positive PDD oversizing (nominal device diameter exceeding the CT-derived PDD) together with an acceptable device-to-basal-RV relationship. Because the native PDD (57.7/58.1 mm) exceeded even the 56-mm device, oversizing was negative (−2.9%/−3.6%), with additionally unfavourable basal-RV oversizing (−16.3%/−3.7%); the manufacturer analysis accordingly classified the anatomy as ‘outside parameters’ with no retention mechanism met (*Table 1*; *Figure 2A*).

**Figure 2:**
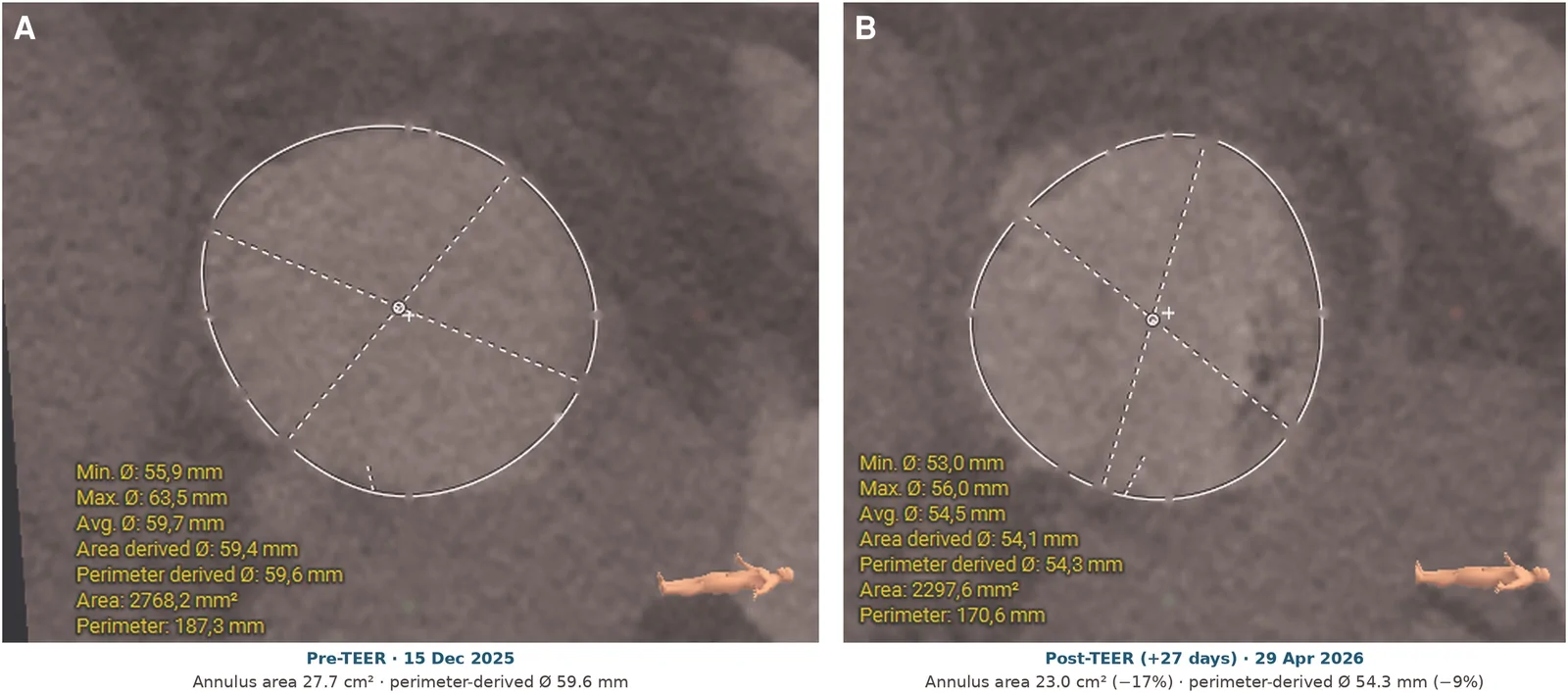
Author-performed CT analysis of the tricuspid annulus before and after the TriArtic procedure. Dedicated, author-performed CT reconstruction of the tricuspid annulus (multiplanar analysis, annular plane). (*A*) Pre-TEER CT (15 December 2025): the traced annular contour yields an annulus area of 27.7 cm^2^, area-derived diameter of 59.4 mm, and perimeter-derived diameter of 59.6 mm (minimum/maximum diameter of 55.9/63.5 mm)—exceeding the sizing window of the largest (56 mm) EVOQUE valve. (*B*) Post-TEER CT (29 April 2026, 27 days after TriClip): annulus area reduced to 23.0 cm^2^ (−17%), area-derived diameter to 54.1 mm, and perimeter-derived diameter to 54.3 mm (minimum/maximum 53.0/56.0 mm). The TriClip itself grasps the leaflet bodies apical to the annular contouring plane and is therefore not visualized at this level; its position at the septo-anterior commissure is shown in *Figure 3*. These independent author measurements differ marginally from the manufacturer screening values (*Table 1*) owing to cardiac-phase selection and contouring methodology, but concordantly demonstrate conversion of the annulus from outside- to within-parameters anatomy for the 56 mm EVOQUE. TEER, transcatheter edge-to-edge repair.

**Table 1:** Key tricuspid CT screening parameters before and 27 days after the TriArtic procedure (single commissural TriClip)

Parameter	Baseline CT (15 December 2025)	Repeat CT +27 days (29 April 2026)	Change
Annulus area (cm^2^)	25.8	22.9	−11%
PDD, diastole/systole (mm)	57.7/58.1	54.2/54.0	≈ −6%
Basal-RV short-axis diameter (mm)	71.7/62.3	65.2	—
PDD oversizing, 56 mm valve (%)	−2.9/−3.6	+3.3/+3.7	—
Basal-RV oversizing (%)	−16.3/−3.7	−8.0/ + 10.1	—
Optimal valve size	N/A	56 mm	—
Manufacturer classification	Outside parameters (no retention mechanism)	Within parameters with significant considerations	—

CT, computed tomography; PDD, projected derived diameter; RV, right ventricle.

Oversizing values are given for diastole/systole and refer to the 56-mm EVOQUE valve; positive values indicate the device is larger than the native annulus (required for anchoring). This author-generated table summarizes the manufacturer-derived screening measurements; an independent, author-performed CT re-analysis of both datasets is shown in *Figure 2*.

After multidisciplinary Heart Team review (10 March 2026), standalone T-TEER was judged inadequate for durable coaptation in this massively dilated annulus, and standalone EVOQUE remained ineligible (*Figure 3*). Given the prohibitive surgical risk, the absence of any established standalone transcatheter option and symptoms refractory to optimized therapy, the Heart Team proposed a sequential, individualized strategy—the TriArtic procedure—comprising transcatheter annuloplasty by commissural TriClip implantation followed, after CT re-screening, by EVOQUE implantation. The Heart Team explicitly recognized that this sequential construct is a novel, non-established, and effectively off-label combination of two individually approved devices, undertaken with the specific goal of rendering EVOQUE feasible. The strategy—including its experimental nature, the anticipated procedural challenges, and the associated risks—was discussed with the patient at a dedicated bedside Heart Team visit, and written informed consent for the individualized off-label approach was obtained and documented.

**Figure 3:**
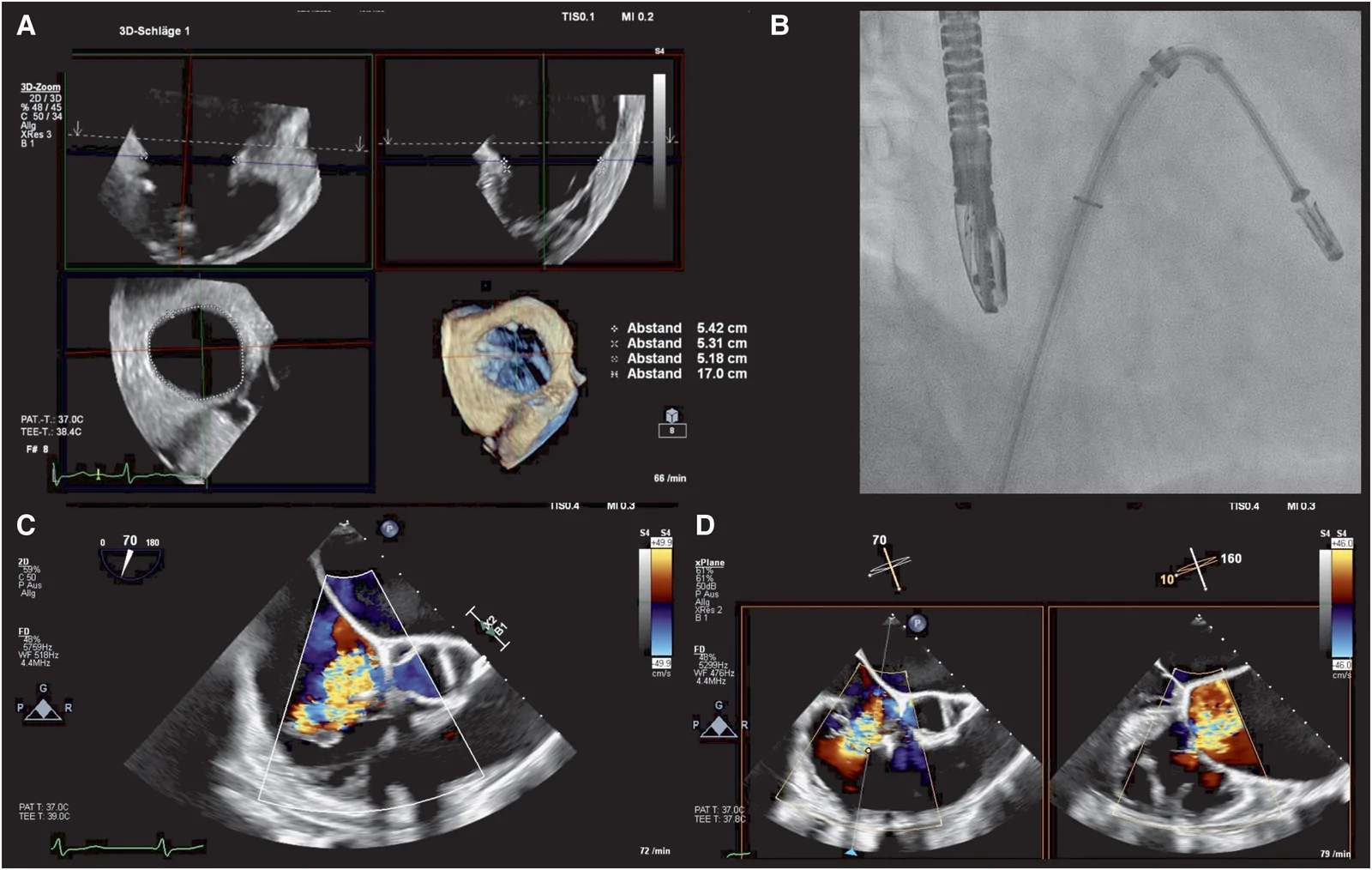
The TriArtic procedure. The TriArtic procedure (2 April 2026): a single TriClip XTW deployed in the septo-anterior (SA) position adjacent to the SA commissure as the transcatheter annular-reduction step. (*A*) Pre-procedural 3D transoesophageal en face view of the dilated tricuspid valve. (*B*) Procedural fluoroscopy showing the steerable guide and TriClip delivery system in the SA position. (*C*) Post-deployment colour-Doppler transoesophageal echocardiography demonstrating reduced tricuspid regurgitation. (*D*) Post-deployment biplane colour-Doppler view confirming the reduction across two orthogonal planes. Awake transthoracic echocardiography subsequently confirmed moderate residual TR (EROA 0.28 cm^2^, vena contracta 6.5 mm) with a mean transtricuspid gradient of 1 mm Hg. SA = septo-anterior; TR = tricuspid regurgitation.

On 2 April 2026, elective TriClip implantation was performed under analgosedation via transfemoral venous access (puncture-to-suture 20 min; fluoroscopy 12.4 min; dose–area product 1869.8 cGy·cm^2^). A TriClip XTW device was selected in preference to a narrower clip or a PASCAL implant on anatomical grounds: The wide (‘W’) arms of the XTW maximize leaflet capture and the amount of intercommissural tissue drawn together per clip, thereby maximizing the intended annuloplasty (annular-downsizing) effect across a large coaptation gap; the independent leaflet-grasping mechanism and steerable delivery system facilitated precise commissural positioning; and the device was readily available with established operator familiarity. A single clip was deployed in a septo-anterior (SA) position adjacent to the SA commissure—the segment with the largest residual leaflet excursion and the most favourable grasping substrate, and the position offering the greatest geometric lever on the septolateral dimension that principally determines the PDD. The septo-posterior commissure was not selected, in order to avoid encroaching on the intended EVOQUE anchoring zone and RV inflow. A single device was chosen deliberately: the targeted annular reduction was achieved with one clip, whereas additional clips would consume leaflet tissue, raise the transvalvular gradient, and risk a double-orifice configuration that could complicate subsequent EVOQUE anchoring and deployment; a second clip was considered but withheld once the target reduction was confirmed.

Intraprocedural transoesophageal echocardiography under analgosedation documented stable clip position with persistent grade IV TR and a mean transtricuspid gradient of 1 mm Hg; no procedural complications occurred. Awake transthoracic echocardiography 5 days later confirmed a meaningful reduction (EROA 0.28 cm^2^, vena contracta 6.5 mm, regurgitant volume 28 mL, mean gradient 1 mm Hg) and a parallel reduction of the concomitant mitral regurgitation to mild. The patient was discharged after 6 days (1–7 April 2026) in NYHA class II–III on her unchanged guideline-directed regimen (sacubitril/valsartan, bisoprolol, spironolactone, empagliflozin, and furosemide 40 mg once daily) with structured outpatient loop-diuretic optimization. During the interval before EVOQUE, renal function remained stable (27 days after TriClip: serum creatinine 0.97 mg/dL, eGFR 59 mL·min^−1^·1.73 m^−2^; cystatin-C–derived eGFR 54 mL·min^−1^·1.73 m^−2^); the post-TEER interval NT-proBNP obtained the day before EVOQUE (19 May 2026) was 1420 pg/mL, reflecting interim volume re-accumulation that prompted preprocedural decongestion (see below).

Repeat EVOQUE screening CT, 27 days after TriClip (29 Apr 2026), demonstrated a substantially smaller annulus (area 22.9 cm^2^; PDD 54.2/54.0 mm—an ∼11% reduction in annular area and ∼6% reduction in PDD relative to baseline; basal-RV short-axis diameter 65.2 mm). Manufacturer reanalysis reclassified the anatomy as ‘within parameters with significant considerations’ (optimal valve size 56 mm; PDD oversizing +3.3%/+3.7%; basal-RV oversizing −8.0%/+10.1%), with the intact TriClip identified at the SA commissure (*Table 1*). An independent, author-performed re-analysis of both CT datasets confirmed the annular reduction (annulus area 27.7 → 23.0 cm^2^; perimeter-derived diameter 59.6 → 54.3 mm; *Figure 2*), concordant with the manufacturer analysis. Because acceptance now rested on a modest positive PDD-oversizing margin—a dimension that enlarges with volume loading—preprocedural euvolaemia was regarded as a principal safeguard against loss of the achieved anchoring margin. Euvolaemia was targeted and confirmed clinically (jugular venous pressure, peripheral oedema and daily weight to an individualized dry weight), by echocardiographic inferior-vena-cava assessment and biochemically, through structured outpatient diuretic titration.

On 20 May 2026—48 days (approximately 7 weeks) after TriClip—a transfemoral 56 mm EVOQUE valve (SN 2113169048) was implanted under analgosedation (procedure time 25 min; fluoroscopy 14 min; dose–area product 1895.6 cGy·cm^2^), with both TriClip anchor loops engaged within the device frame. Immediately before the procedure, transient type-1 cardiorenal deterioration was observed (serum creatinine 1.44 mg/dL, eGFR 36 mL·min^−1^·1.73 m^−2^; NT-proBNP 1420 pg/mL) in the context of interval congestion, prompting preprocedural decongestion. Post-implant assessment documented stable valve position with TR grade 0, a mean transtricuspid gradient of 1 mm Hg, and no paravalvular leak (*Figure 4*). Predischarge transthoracic echocardiography (26 May 2026) confirmed an excellent result with no significant residual regurgitation and a mean gradient of 1 mm Hg (*Figure 5*); TAPSE decreased from 25 to 15 mm (see Discussion).

**Figure 4:**
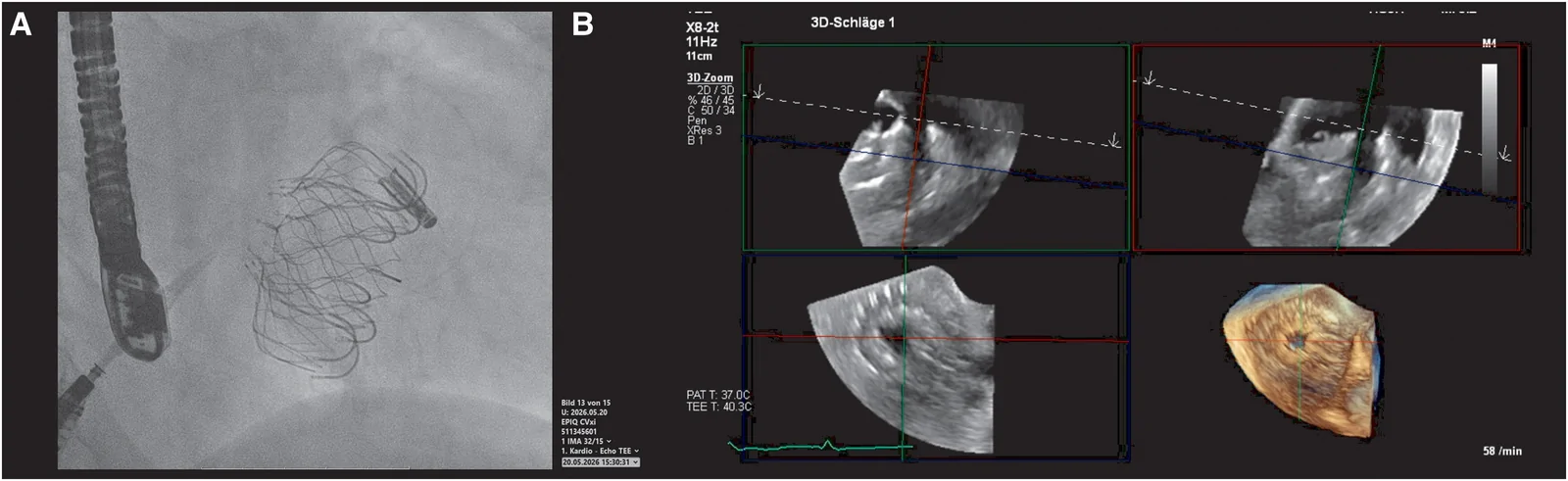
EVOQUE 56 mm transfemoral implantation. Transfemoral implantation of a 56-mm EVOQUE valve under analgosedation (20 May 2026). (*A*) Fluoroscopy after deployment showing the expanded EVOQUE nitinol frame in the tricuspid position with the pre-existing TriClip incorporated within the construct. (*B*) Post-implant 3D transoesophageal en face view demonstrating a well-seated valve. The staged TriArtic construct was thereby completed.

**Figure 5:**
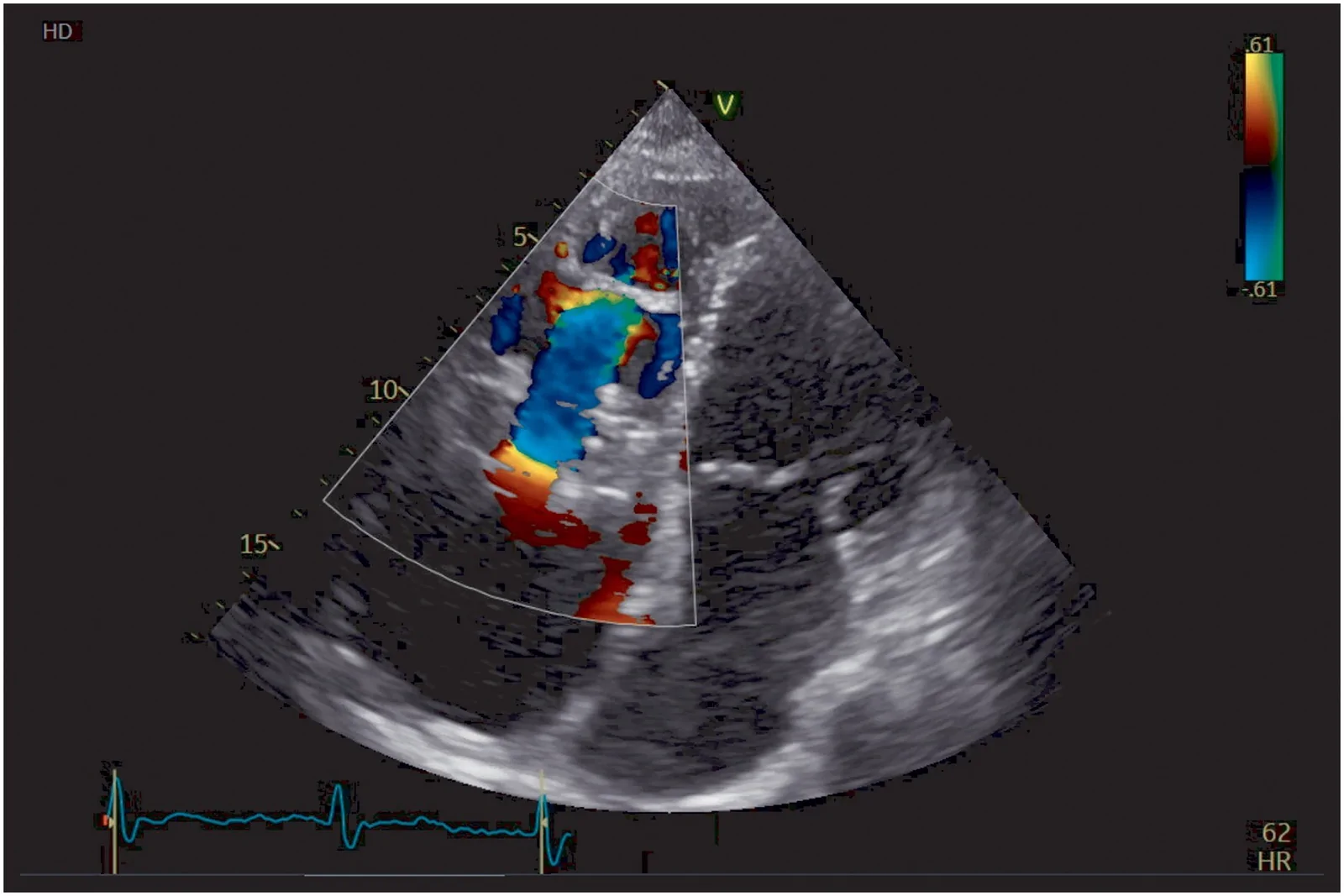
Post-EVOQUE transthoracic echocardiography. Predischarge post-EVOQUE transthoracic colour-Doppler echocardiography (26 May 2026) demonstrating the EVOQUE valve *in situ* with no significant residual tricuspid regurgitation and a mean transtricuspid gradient of 1 mm Hg. TAPSE was 15 mm (reduced from 25 mm at baseline); see Discussion for interpretation.

## Outcome

Postoperative recovery was uneventful. NT-proBNP declined on the day of the EVOQUE procedure from 1420 to 968 pg/mL despite an expected periprocedural rise in high-sensitivity troponin T (19 → 81 ng/L after right-heart device deployment). Estimated glomerular filtration rate recovered rapidly from 36 mL·min^−1^·1.73 m^−2^ immediately preprocedure to 69 mL·min^−1^·1.73 m^−2^ within 24 h, with normalization of serum creatinine (0.85 mg/dL)—confirming the reversible, congestion-related (type-1 cardiorenal) nature of the pre-EVOQUE deterioration rather than intrinsic chronic kidney disease. Bilirubin remained normal and γ-glutamyltransferase fell over the post-EVOQUE course. The patient was discharged in NYHA functional class II after 7 days (19–26 May 2026) with marked symptomatic improvement.

## Discussion

This case illustrates an increasingly recognized ‘anatomical dead zone’ in transcatheter tricuspid intervention: an annulus too dilated for current TTVR sizing matrices yet unlikely to achieve durable coaptation with standalone T-TEER. With broader EVOQUE availability after TRISCEND II,^1^ structured CT screening^5^ identifies a non-trivial proportion of otherwise-suitable candidates as ‘outside parameters’, predominantly because the annular area or PDD exceeds even the largest (56 mm) device. Because manufacturer ‘within-parameters’ acceptance can depend on a small positive PDD-oversizing margin as the principal retention mechanism, any preprocedural annular reduction is proportionately impactful—and, conversely, is volume dependent and must be protected by euvolaemia.

The TriArtic procedure is, in effect, a transcatheter tricuspid annuloplasty: a single commissural TriClip shortens the annular long axis, reduces overall area, and may improve the device-to-basal-RV relationship. In this patient, a single TriClip XTW in the SA position produced an 11% reduction in annular area and a 6% reduction in PDD and—decisively—converted the manufacturer verdict from outside to within parameters. The concept parallels the ARCTIC technique for mitral annular calcification, in which TEER is used not primarily to treat regurgitation but to mechanically downsize an annulus otherwise unsuitable for the intended transcatheter valve.^4^ Current TR management frameworks (the 2021 ESC/EACTS guidelines and the EHJ atrial-secondary-TR position paper) address individual device candidacy but do not yet recognize sequential transcatheter annuloplasty followed by valve replacement as a defined pathway.^6,7^

A clinically valuable consequence of the TriClip was incremental TR reduction (torrential → moderate; EROA 2.1 → 0.28 cm^2^), which permitted the 48-day (≈7-week) interval of clinical stabilization, outpatient diuretic optimization, and cardiorenal recovery before EVOQUE. The transient discrepancy between the intraprocedural assessment under analgosedation (residual grade IV) and the awake echocardiogram 5 days later (moderate TR) reflects the well-described loading-condition dependency of TR grading.

An additional determinant of the favourable outcome deserves emphasis. From a clinical standpoint, this patient was treated at a comparatively early stage of the disease trajectory: right-ventricular systolic function was preserved (TAPSE 25 mm), invasive haemodynamics excluded significant pulmonary hypertension, baseline renal function was preserved, and the pre-procedural cardiorenal deterioration was congestion-related and fully reversible. Earlier rather than later referral is increasingly recognized as a key determinant of post-procedural outcome in transcatheter tricuspid intervention, as irreversible right-ventricular remodelling, fixed pulmonary vascular disease, and established end-organ dysfunction attenuate the benefit of even technically successful TR elimination.^8,9^ The excellent final result in this case should therefore not be attributed to the annular-downsizing strategy alone: careful patient selection—preserved right-ventricular reserve, absence of significant pulmonary hypertension, and reversible congestion/cardiorenal physiology—very likely contributed substantially, and these selection criteria should be regarded as integral to, rather than separable from, the staged TriArtic concept.

The fall in TAPSE from 25 to 15 mm after EVOQUE most likely reflects the altered RV loading conditions that follow abrupt elimination of a low-impedance regurgitant pathway, whereby RV afterload increases and the longitudinal contribution to shortening is unmasked. However, load-dependent longitudinal indices such as TAPSE are difficult to interpret after TTVR, and an early contractile component cannot be excluded; this change should therefore be interpreted cautiously and monitored on serial imaging rather than attributed definitively to a single mechanism.

The concomitant reduction of mitral regurgitation to mild was temporally associated with the intervention. While left-atrial unloading and a reduction in interatrial and pericardial constraint may have contributed, any reverse-remodelling mechanism over this short interval is speculative, and load-dependent measurement variability may account for part of the observed change; this association is therefore reported cautiously and without a definitive mechanistic claim.

Several procedure-specific hazards of implanting EVOQUE into a valve that already carries a TriClip warrant emphasis. During valve crossing and deployment, guidewires and the delivery system may become entrapped in the clip or its anchor loops; the clip may be dislodged or embolized; and interaction between the clip and the expanding nitinol frame may produce incomplete or asymmetric frame expansion, malposition, or paravalvular leak. In the present case, both anchor loops were deliberately captured within the frame and post-implant imaging excluded paravalvular leak, but these hazards are real and mandate meticulous multimodality imaging guidance and preparedness for bail-out.

Longer-term, the durability of the staged construct is unknown. The post-TriClip anatomy remained ‘within parameters with significant considerations’ with only a modest positive oversizing margin; in such borderline anatomy, the risk of late valve migration or dislodgement—potentially aggravated by volume-dependent annular re-dilatation or by a late change in the clip–frame relationship—cannot be excluded and warrants close, protocolized surveillance.

This report has important limitations. It describes a single, highly selected patient, and the memorable label ‘TriArtic procedure’ should not be read as establishing a new, generally applicable procedural category; it denotes feasibility in this individual rather than validated efficacy or safety. The geometric benefit of commissural TEER is strongly position-dependent—a SA clip was advantageous here, but other positions could compromise EVOQUE anchor geometry—and both procedures still required ‘yellow’-tier acceptance contingent on strict volume-status optimization. Favourable selection factors in this case—early-stage disease with preserved right-ventricular function (TAPSE 25 mm), absence of significant pulmonary hypertension, and reversible cardiorenal physiology—very likely contributed to the outcome independently of the annular-reduction strategy itself. The sequential strategy is a novel, non-established and effectively off-label use of two individually approved devices; it was undertaken only after explicit Heart Team deliberation and documented patient consent, and it should be reserved for comparable high-risk patients without an established standalone option. Prospective, multicentre experience with systematic imaging follow-up is required to define selection criteria, optimal clip position and long-term durability.

## Conclusions

In a high-risk patient with torrential atrial functional TR, HFrEF and a tricuspid annulus initially rejected as ‘outside parameters’ for EVOQUE TTVR, a single commissural TriClip XTW deployed as a transcatheter annuloplasty step converted the annulus into within-parameters anatomy and enabled subsequent successful transfemoral EVOQUE implantation. In carefully selected patients for whom no standalone transcatheter option exists, sequential transcatheter tricuspid annuloplasty followed by valve replacement may be feasible; this single-case experience does not establish general applicability, and prospective study is needed to define selection criteria, optimal clip position, and long-term durability.

## Lead author biography


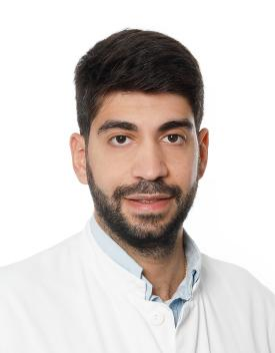
Raphael Phinicarides, MD completed his medical studies at the Heinrich Heine University Düsseldorf, Germany, and is currently a resident at the Department of Cardiology, Pulmonology, and Vascular Medicine, University Hospital Düsseldorf. His clinical and research interests focus on transcatheter structural heart interventions, advanced heart failure and heart transplantation.

## Data Availability

The data underlying this case report are available from the corresponding author upon reasonable request, subject to institutional data-protection requirements.
